# Comparative Efficacy of Monotherapies for Chronic Hand Eczema: A Network Meta‐Analysis Study

**DOI:** 10.1111/jocd.70552

**Published:** 2025-11-24

**Authors:** Aditya K. Gupta, Mary A. Bamimore, Mesbah Talukder

**Affiliations:** ^1^ Mediprobe Research Inc. London Ontario Canada; ^2^ Division of Dermatology, Department of Medicine University of Toronto School of Medicine Toronto Ontario Canada; ^3^ School of Pharmacy BRAC University Dhaka Bangladesh

## Abstract

**Introduction:**

Chronic hand eczema (CHE) is a highly prevalent dermatology‐related occupational hazard. This condition, which is a distinct diagnosis from atopic dermatitis (AD), can be quite symptomatic with implications for one's personal and professional life. Many therapeutic interventions exist for CHE, and the current study determined—through network meta‐analyses (NMAs)—the relative efficacy of the newer treatments for CHE.

**Methods:**

Data for eligible studies were identified after systematically reviewing the literature in PubMed and Scopus. Eligible studies were those that had an arm investigating the impact of monotherapy on 12‐ or 16‐week changes in Hand Eczema Severity Index (HECSI) scores. We conducted sensitivity analyses where network meta‐regressions were performed to (ecologically) adjust for variation due to age and sex. Base and sensitivity analyses were adjusted for disease severity at baseline.

**Results:**

We identified 5 eligible studies—across which there were 10 active comparators. Among the active comparators were two treatments approved by the Food and Drug Administration (FDA) and European Medicines Agency (EMA) for the treatment of CHE, namely, alitretinoin 30 mg once daily (oral) and delgocitinib 20 mg/g twice daily (topical). We estimated pairwise relative effects using the mean difference (i.e., mean reduction) in the respective HECSI scores; furthermore, efficacy was ranked with Surface Under the Cumulative Ranking Curve (SUCRA) values.

**Conclusions:**

We attempted to contribute to the existing CHE literature by producing comparative information on its treatments' relative efficacy; moreover, we found no published NMA study on available treatments for CHE before the conduct of our work. Our results can guide clinical decision‐making.

## 1 Introduction

Chronic hand eczema (CHE) is an inflammatory condition that often spells occupational, psychological, and functional challenges [1]. Persistence of hand eczema for over 3 months—or the occurrence thereof at least twice a year—confirms a diagnosis of CHE [1, 2]. Though CHE is a distinct diagnosis from atopic dermatitis (AD), the two sometimes co‐occur [3]; moreover, a diagnosis of AD in itself is a risk factor for the development of CHE [4]. Other risk factors include early age of onset, low education level, nicotine consumption, and mutations in the filaggrin (FLG) gene [4].

Symptomatically, itch and pain are classical hallmarks of CHE—the two of which are reasons numerous efficacy studies for CHE use itch and pain scores as endpoints [2]; the Hand Eczema Severity Index (HECSI) is a validated outcome measure for CHE [5], and numerous trials have used it [6, 7].

In many countries, CHE is the most common occupational disease—where 19.9% and 23% of sick leaves and job loss, respectively, are attributable to CHE [7]. Various treatments have been formulated to manage this disease and evidence on the comparative effect thereof is scant. We conducted a network meta‐analysis (NMA) study to determine the relative efficacy of monotherapies for CHE.

## 2 Methods

The protocol for the current study was registered on the Open Science Framework (OSF) platform—and can be viewed via the following link: https://doi.org/10.17605/OSF.IO/36M92 [8]; the conduct of our work followed the Preferred Reporting Items for Systematic Reviews and Meta‐Analyses (PRISMA) guidelines for NMAs [9]. All analyses were conducted with *R* software via *RStudio* [10]. In all analyses, the threshold for statistical significance was 5% or 0.05.

Studies whose data were to be used for quantitative analyses were identified through a systematic search of the literature through PubMed, Scopus, and reference mining. The entire search process was independently conducted by two authors (M.B. and M.T.); any disagreements during the screening of titles, abstracts, or full texts were resolved through discussion with a third author (A.K.G.). The systematic search was managed using Rayyan software [11], and extracted data were managed using spreadsheets.

As per the PICO (i.e., patient, intervention, comparator, and outcome) framework, studies whose data would be eligible for quantitative analyses were those that investigated the impact of monotherapies on CHE insofar as the mean change in HECSI [12] scores between 12 and 16 weeks from baseline. This outcome measure was chosen because it is a popular, validated tool for measuring CHE [13]. In addition to our PICO, eligible studies had to be randomized and published in English. We qualitatively assessed eligible studies' quality of evidence using a revised Risk of Bias (RoB) tool and presented our evaluations with a traffic plot [14].

We used network plots to depict the geometry of our networks—one per outcome. A network plot is a graph of nodes and edges where a node corresponds to a comparator; an edge represents the comparison of two nodes whose effect was determined in an actual head‐to‐head trial; in other words, a network plot picturizes direct evidence. An NMA has the ability to quantify indirect evidence, i.e., estimate the relative effect of comparators that were not actually compared in a trial study. Consistency between direct and indirect evidence can be determined through node‐splitting analysis, given that the network has a closed loop—where closed loops from a multi‐arm trial are excluded [15].

We conducted a Bayesian NMA under a fixed‐effects model with uniform priors. For sensitivity analyses, we adjusted the base NMA for variation in age and sex. All NMA models (i.e., base and sensitivity analyses) were adjusted for disease severity at baseline. The adjustments of age, sex, and severity were done ecologically, i.e., using aggregate‐level data—as opposed to individual patient data. Each Bayesian NMA produced estimates of each comparator's Surface Under the Cumulative Ranking Area (SUCRA) value, a metric that ranks the efficacy of 3 or more interventions. The value of SUCRA ranges from 0 to 1, or 0% to 100% (inclusive). An NMA also produces point estimates of relative efficacy for every possible pairwise comparison—and such comparisons are presented in “league tables,” where each cell therein shows the mean difference (MD) (as in mean reduction) and 95% credible intervals (CI).

## 3 Results

**FIGURE 1:**
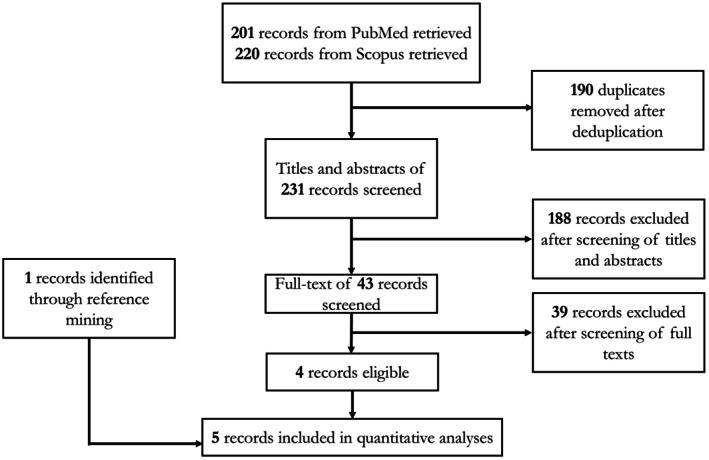
Identification of included studies.

**FIGURE 2:**
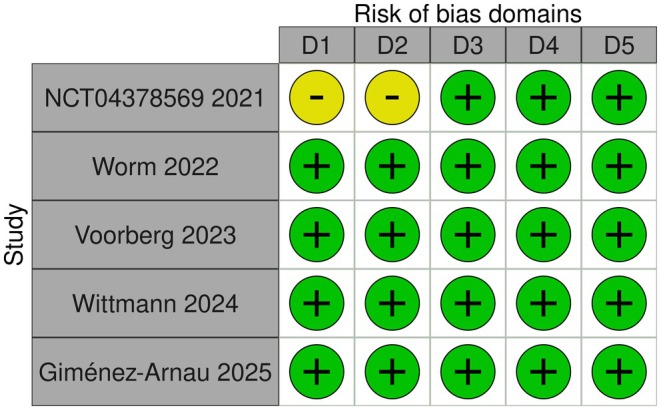
A traffic plot generated by Cochrane's Collaboration's Risk of Bias tool. D1 represents bias due to the randomization process, D2 represents bias through deviations from planned intervention, D3 represents bias arising from missing outcome data, D4 represents bias in measurement of the outcome, and D5 represents bias in selection of the reported result.

**FIGURE 3:**
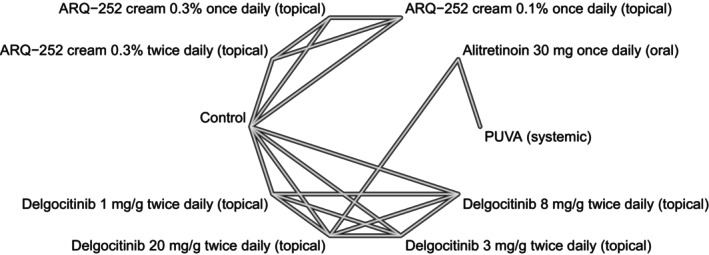
A network plot for our 12‐week change in Hand Eczema Severity Index (HECSI) scores for oral (i.e., systemic) and topical (i.e., local) monotherapies.

**FIGURE 4:**
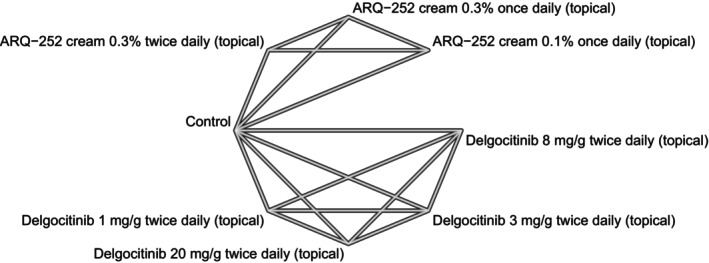
A network plot for our 12‐week change in Hand Eczema Severity Index (HECSI) scores for topical (i.e., local) monotherapies.

**FIGURE 5:**
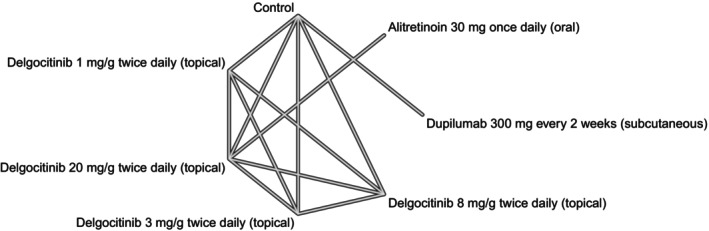
A network plot for our 16‐week change in Hand Eczema Severity Index (HECSI) scores for oral (i.e., systemic) and topical (i.e., local) monotherapies.

Our systematic search identified 5 randomized trials [16, 17, 18, 19, 20] whose data met our eligibility criteria for quantitative analyses (Figure 1). A summary of the eligible trials' study characteristics is presented in Table 1; qualitative evaluation of each trial's evidence quality is presented in Figure 2. The available data were sufficient to conduct NMAs for three specific outcomes, namely, (1) 12‐week mean change in HECSI scores for only topical comparators, (2) 12‐week mean change in HECSI scores for both topical and oral (or systemic) comparators, and (3) 16‐week mean change in HECSI scores for both topical and oral (or systemic) comparators. The geometry of each outcome's network is depicted in Figures 3, 4, 5.

**TABLE 1:** Table of characteristics.

Reference	Arms	*N*	Age in years (mean, SD)	Sex (males, females)
NCT04378569 2021	ARQ‐252 cream 0.3% once daily (topical)	65	41.6, 15.08	28, 37
	ARQ‐252 cream 0.3% twice daily (topical)	61	43.3, 15.05	22, 39
	ARQ‐252 cream 0.1% once daily (topical)	32	41.6, 15.33	11, 21
	Vehicle	65	41.8, 12.7068	9, 23
Worm 2022	Delgocitinib 1 mg/g twice daily (topical)	52	44.3, 13.6	37, 15
	Delgocitinib 3 mg/g twice daily (topical)	51	46.1, 14.6	28, 23
	Delgocitinib 8 mg/g twice daily (topical)	52	47.9, 12.9	32, 20
	Delgocitinib 20 mg/g twice daily (topical)	53	43.9, 15.1	34, 19
	Vehicle	50	47.8, 16.2	27, 23
Voorberg 2023	Dupilumab 300 mg every 2 weeks (subcutaneous)	20	44.5, 15.1	11, 9
	Placebo	9	42.8, 14.4	3,6
Wittmann 2024	PUVA (systemic)	218	45.1, 15.2	141, 77
	Alitretinoin 30 mg once daily (oral)	217	46.5, 14.9	132, 85
Giménez‐Arnau 2025	Delgocitinib 20 mg/g twice daily (topical)	254	46, 5.5	87 167
	Alitretinoin 30 mg once daily (oral)	259	44, 6.25	92 167

**FIGURE 6:**
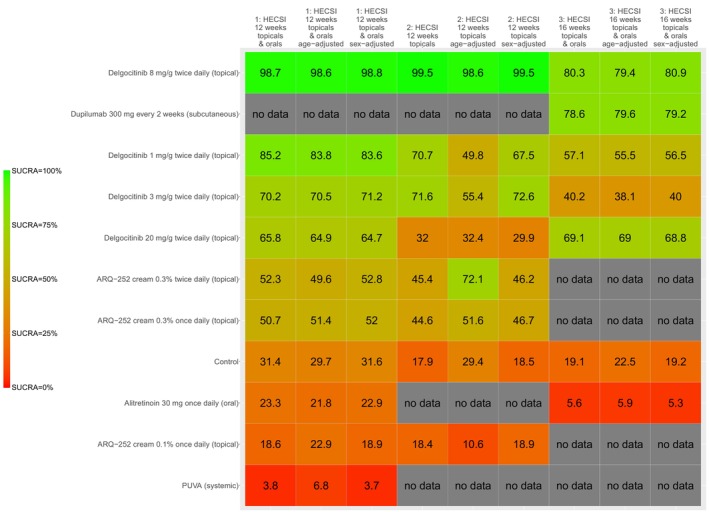
Understanding the Surface Under the Cumulative Ranking Curve (SUCRA) on a kilim plot. The SUCRA value of a comparator reflects its overall efficacy ranking; higher values indicate a more favorable effect. The vertical axis represents the comparators—which are a total of 10 active comparators and one inactive control (i.e., the placebo/vehicle node). The horizontal axis displays the respective endpoints (from base and sensitivity analyses) and the respective SUCRA values are represented as percentages. The colors red and green (selected arbitrarily) denote the lowest and highest SUCRA values, respectively. This visual aid illustrates treatments ranked from least to most effective based on this metric.

**FIGURE 7:**
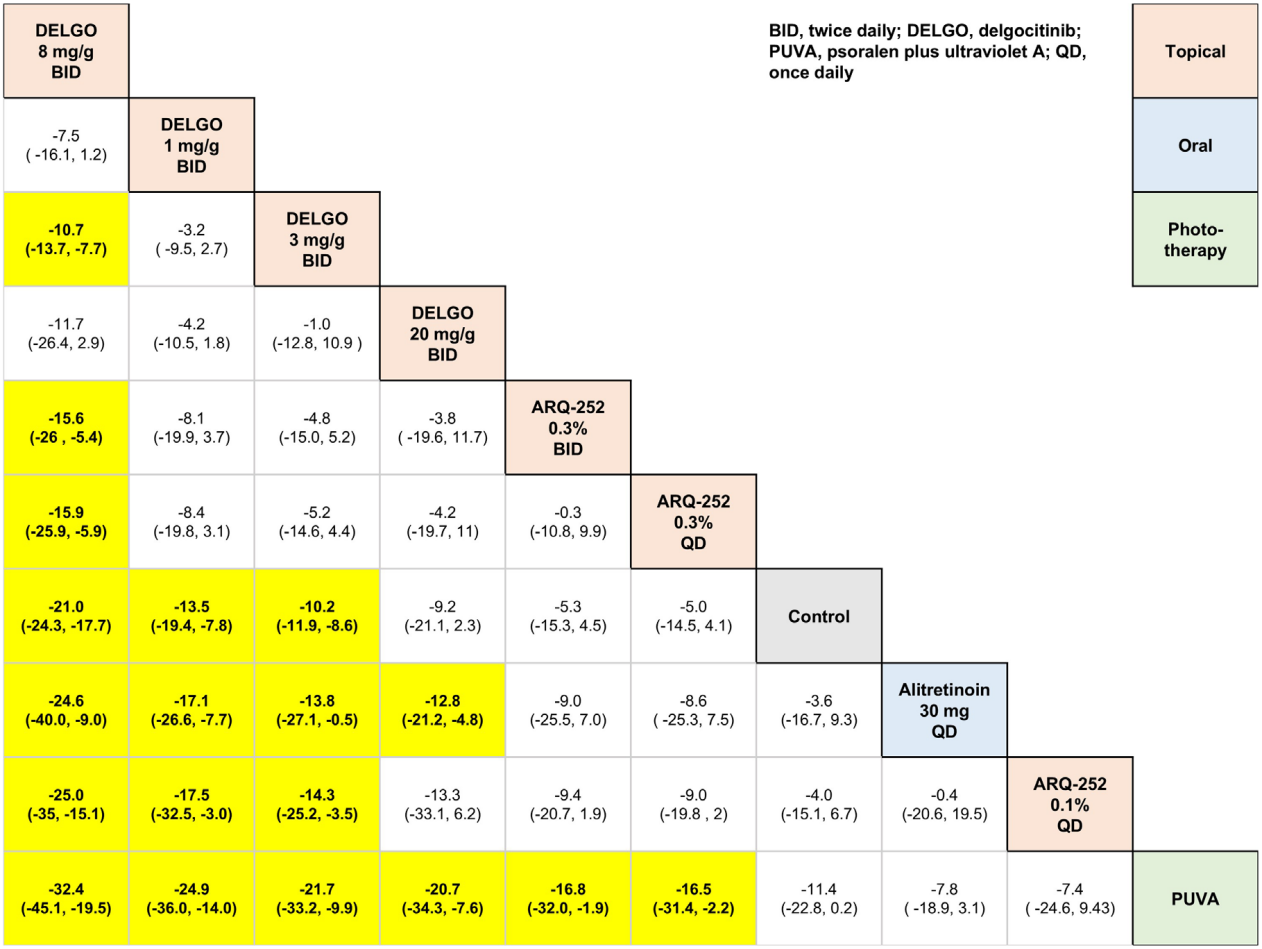
League table for 12‐week change in Hand Eczema Severity Index (HECSI) scores for oral (i.e., systemic) and topical (i.e., local) monotherapies.

**FIGURE 8:**
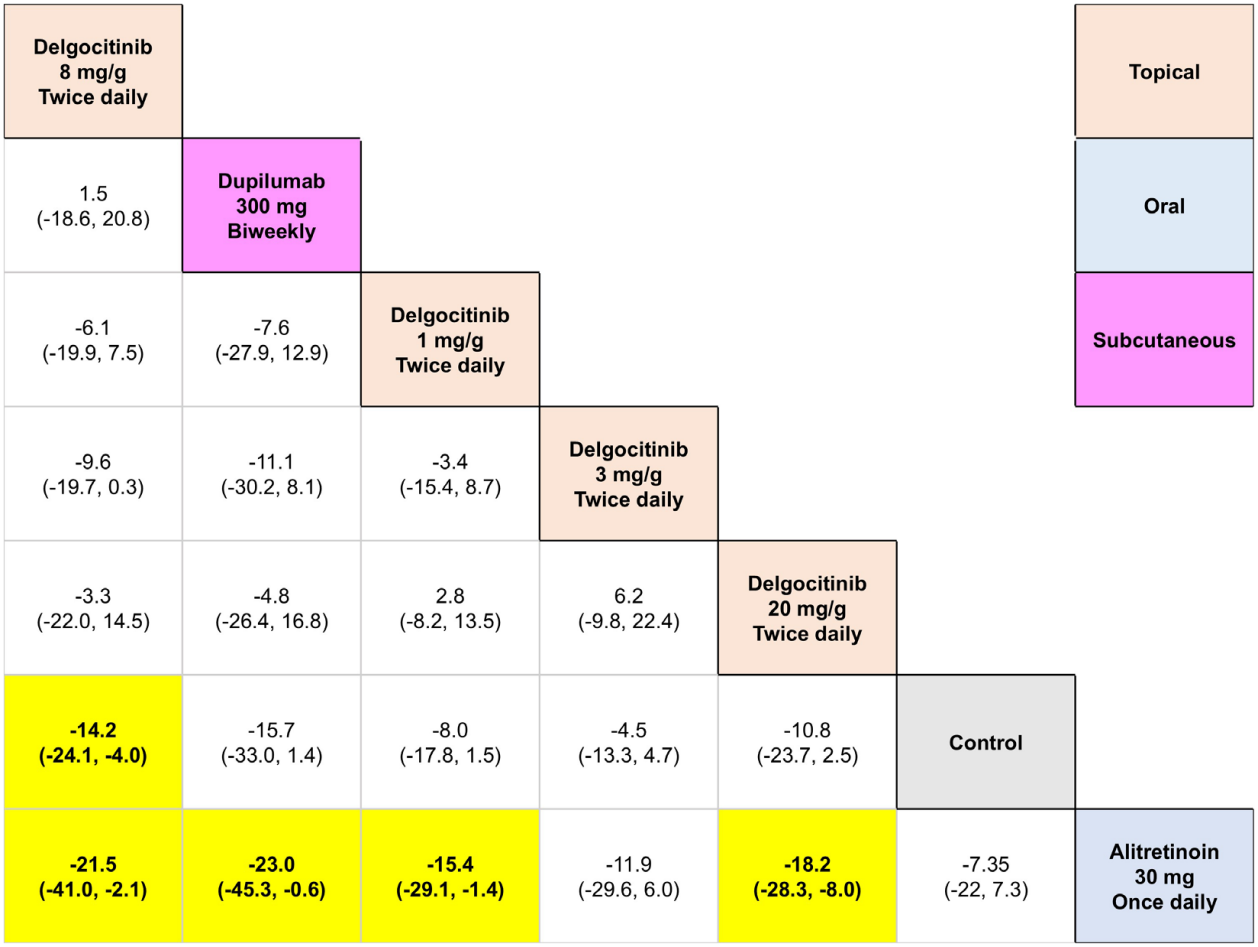
League table for 16‐week change in Hand Eczema Severity Index (HECSI) scores for oral (i.e., systemic) and topical (i.e., local) monotherapies.

The kilim plot in Figure 6 presents all interventions' SUCRA values across the base and sensitivity analyses. Across the three networks, 10 active comparators were identified, namely, (1) Alitretinoin 30 mg once daily (oral), (2) ARQ‐252 cream 0.1% once daily (topical), (3) ARQ‐252 cream 0.3% once daily (topical), (4) ARQ‐252 cream 0.3% twice daily (topical), (5) Delgocitinib 1 mg/g twice daily (topical), (6) Delgocitinib 20 mg/g twice daily (topical), (7) Delgocitinib 3 mg/g twice daily (topical), (8) Delgocitinib 8 mg/g twice daily (topical), (9) Dupilumab 300 mg every 2 weeks (subcutaneous), and (10) PUVA (systemic)—where PUVA refers to psoralen and ultraviolet A; ARQ‐252 is a topical Janus kinase (JAK) inhibitor. Across the three networks (i.e., as per the three outcomes), Delgocitinib 8 mg/g twice daily (topical) was ranked the most efficacious—in both the base and sensitivity analyses (Figure 6). League tables are presented in Figures 7 and 8.

Delgocitinib 8 mg/g twice daily (topical) was significantly more efficacious than Alitretinoin 30 mg once daily (oral) in terms of 12‐week mean change in HECSI scores for both topical and oral (or systemic) comparators insofar as the base analysis (MD = 24.65, 95% CI: 9.05, 40.07, *p* < 0.05), sex‐adjusted analysis (MD = 24.24, 95% CI: 9.48, 39.33, *p* < 0.05), and age‐adjusted analysis (MD = 24.91, 95% CI: 9.17, 40.85, *p* < 0.05).

Delgocitinib 8 mg/g twice daily (topical) was significantly more efficacious than alitretinoin 30 mg once daily (oral) in terms of 16‐week mean change in HECSI scores for both topical and oral (or systemic) comparators insofar as the base analysis (MD = 21.56, 95% CI: 2.16, 41.09, *p* < 0.05), sex‐adjusted analysis (MD = 21.59, 95% CI: 1.70, 41.17, *p* < 0.05), and age‐adjusted analysis (MD = 21.52, 95% CI: 2.40, 41.15, *p* < 0.05).

Dupilumab 300 mg every 2 weeks (subcutaneous) was more efficacious than Alitretinoin 30 mg once daily (oral) (MD = 23.06, 95% CI: 0.66, 45.35, *p* < 0.05) in terms of 16‐week mean change in HECSI under the base analyses, but not the sex‐adjusted analysis (MD = 22.73, 95% CI: −0.88, 45.90, *p* ≥ 0.05) or the age‐adjusted analysis (MD = 23.38, 95% CI: −0.48, 46.97, *p* ≥ 0.05).

## 4 Discussion

The current NMA study produced high‐quality comparative evidence on CHE therapies—inclusive of those approved by regulatory agencies such as the United States Food and Drug Administration (FDA) or European Medicines Agency (EMA). Delgocitinib 20 mg/g cream was approved as a treatment for CHE by the FDA on July 25, 2025 [21]—and on September 23, 2024, by the EMA [22]. Another approved treatment for CHE—which was also a comparator in the current study—is Alitretinoin 30 mg once daily (oral). Delgocitinib is a JAK inhibitor, while alitretinoin is a vitamin A derivative.

The therapeutic effects of numerous treatments for CHE have been investigated in many randomized studies, but—as the Cochrane review by Christoffers et al. [23] pointed out—the stark heterogeneity in choice of outcome measures and timepoints used across these studies has precluded meaningful quantitative syntheses. For example, many of these studies did not use any measure related to HECSI, and/or used outcomes quantified at 3 weeks or less. This could also explain why an NMA study on CHE treatments has not been published in the peer‐reviewed literature hitherto. The conduct of the present work occurred at an opportune moment, as high‐quality empirical evidence with common endpoints—such as the mean change in HECSI 12 and 16 weeks from baseline—was already available at the timing of our systematic review. Topical corticosteroids are a therapeutic option for CHE; however, we did not include studies that investigated them in our NMA due to a lack of common comparators. For example, Thaitirarot et al. [24] compared the efficacy of transdermal betamethasone with that of topical betamethasone ointment on patients with CHE using 8‐week change in HECSI, Physician Global Assessment (PGA), and Dermatology Life Quality Index (DLQI). As another example, Khademi et al. [25] compared the efficacies of betamethasone, pumpkin, Eucerin, and almond ointments using a 4‐week change in HECSI and DLQI scores. The efficacy of other classes of agents, such as calcineurin inhibitors and moisturizers, was not compared for the same reason (i.e., lack of common comparators) [1, 4, 6, 7, 26, 27]; for example, Bauer et al. [4] investigated the efficacy of pimecrolimus 1% cream twice daily and vehicle using an 8‐week change in HECSI, DLQI, etc. scores. A trial that compared the efficacy of ruxolitinib 1.5% cream twice daily (topical) (NCT05906628 in ClinicalTrials.gov) with vehicle was excluded as mean change in HECSI scores was used as an outcome measure.

Studies were also excluded on the grounds of study design. For instance, many studies, including those by Branyiczky et al. [26] and Gomez‐Martinez et al. [27] were excluded as they were retrospective.

CHE is a common skin condition with a global prevalence of 10%; the condition is relatively more prevalent in women than in men; in fact, being of the female sex has been reported to be a risk factor for a CHE diagnosis [6]. So, a strength of our analyses is that we provided sex‐adjusted efficacy estimates because the literature supports being of female sex to be a confounder or effect modifier (i.e., moderator) [6]. Another strength is the fact that all analyses were adjusted for baseline severity using a validated tool (i.e., HECSI). Our results can guide the conduct of future studies and clinical decision‐making.

## Author Contributions

Conception of the manuscript was done by Aditya K. Gupta Data analysis was performed by Mary A. Bamimore The manuscript was drafted by Aditya K. Gupta, Mesbah Talukder, and Mary A. Bamimore, substantively edited and revised by Aditya K. Gupta, Mesbah Talukder, and Mary A. Bamimore.

## Ethics Statement

Approval from an ethics board was not required as there was no direct involvement with human participants.

## Consent

The research did not involve direct interaction with human participants; therefore, informed consent was not required.

## Conflicts of Interest

The authors declare no conflicts of interest.

## Data Availability

Research data are not shared.
